# Smoke-Free**–**Home Rules Among Women With Infants, 2004–2008

**DOI:** 10.5888/pcd9.120108

**Published:** 2012-11-08

**Authors:** Falicia A. Gibbs, Van T. Tong, Sherry L. Farr, Patricia M. Dietz, Stephen Babb

**Affiliations:** Author Affiliations: Falicia A. Gibbs, Sherry L. Farr, Patricia M. Dietz, Stephen Babb, National Center for Chronic Disease Prevention and Health Promotion, Centers for Disease Control and Prevention, Atlanta, Georgia.

## Abstract

**Introduction:**

Exposure to secondhand smoke increases risk for infant illness and death. The objective of this study was to estimate the prevalence of complete smoke-free–home rules (smoking not allowed anywhere in the home) among women with infants in the United States.

**Methods:**

We analyzed 2004–2008 data from the Pregnancy Risk Assessment Monitoring System on 41,535 women who had recent live births in 5 states (Arkansas, Maine, New Jersey, Oregon, and Washington). We calculated the prevalence of complete smoke-free–home rules and partial or no rules by maternal smoking status, demographic characteristics, delivery year, and state of residence. We used adjusted prevalence ratios (APRs) to estimate associations between complete rules and partial or no rules and variables.

**Results:**

During 2004–2008, the overall prevalence of complete rules was 94.6% (95% confidence interval [CI], 94.4–94.9), ranging from 85.4% (Arkansas) to 98.1% (Oregon). The prevalence of complete rules increased significantly in 3 states from 2004 to 2008. It was lowest among women who smoked during pregnancy and postpartum, women younger than 20 years, non-Hispanic black women, women with fewer than 12 years of education, women who had an annual household income of less than $10,000, unmarried women, and women enrolled in Medicaid during pregnancy.

**Conclusion:**

The prevalence of complete smoke-free–home rules among women with infants was high overall and increased in 3 of 5 states, signifying a public health success. Sustained and targeted efforts among groups of women who are least likely to have complete smoke-free–home rules are needed to protect infants from exposure to secondhand smoke.

## Introduction

Infants and children exposed to secondhand smoke are at increased risk for acute respiratory infections, asthma attacks, ear infections, and sudden infant death syndrome (1,2). Despite declines in the prevalence of adult smoking (3) and smoking after delivery among US women with infants (4), exposure to secondhand smoke among infants and children is high. Approximately 32 million US children and adolescents aged 3 to 19 years were exposed to secondhand smoke during 2007 and 2008 (5). Among children and adolescents living with someone who smoked inside the home, almost all (98.3%) had detectable cotinine levels in their blood, indicating exposure to secondhand smoke (5). Although 25 states and the District of Columbia have implemented laws that completely eliminate smoking in public places and workplaces, including restaurants and bars (6), these laws do not extend to the home, a primary source of secondhand smoke exposure for infants and children (2,7). A complete smoking ban in the home decreases secondhand smoke exposure among children (2,7,8). To our knowledge, no population-based studies have examined the prevalence and potential correlates of home smoking rules among women with infants in the United States.

The objective of this study was to estimate the prevalence of complete smoke-free–home rules (smoking not allowed anywhere in the home) among women with infants in the United States. We also assessed the independent associations between complete rules and selected maternal and infant characteristics. A secondary objective was to examine whether women who had partial or no home rules attended a maternal postpartum check-up, attended well-baby visits, or participated in the Special Supplemental Nutrition Program for Women, Infants and Children (WIC). These data could be used to encourage adoption of complete rules among families who have infants at risk for secondhand smoke exposure.

## Methods

### Study design and sample

We analyzed 2004–2008 data from the Pregnancy Risk Assessment Monitoring System (PRAMS). PRAMS is an ongoing population-based surveillance system of maternal behaviors and experiences before, during, and after pregnancy. PRAMS is conducted by state health departments in collaboration with the Centers for Disease Control and Prevention (CDC). All state health departments participating in PRAMS use a standardized data collection methodology developed by CDC. Each month, a stratified sample of approximately 100 to 300 mothers who delivered live infants is selected from birth certificate records in each participating state. A questionnaire is mailed to mothers postpartum; nonresponders receive telephone follow-up. To minimize recall bias, efforts are made to contact women within 9 months of delivery. Survey data are linked to birth certificate data and weighted for sample design, nonresponse, and noncoverage to represent all women delivering a live birth in each state. Detailed methodology of PRAMS is available elsewhere (9).

We included PRAMS data from 5 states (Arkansas, Maine, New Jersey, Oregon, and Washington) in this analysis; we chose these states because they included a state-option question on smoke-free–home rules. Annual weighted response rates by state ranged from 71% to 82%. For these years, women participated in the survey an average of 3.5 months after delivery.

### Variables

Smoke-free–home rules were determined by asking the following question: “Which of the following statements best describes the rules about smoking inside your home now?” Respondents were categorized as having complete rules (“no one was allowed to smoke anywhere inside my home”); partial rules (“smoking was allowed in some rooms or at some times”); or no rules (“smoking was permitted anywhere inside my home”). The survey did not ask respondents whether or how these rules were enforced.

We examined the following maternal demographic characteristics: postpartum smoking status, age, race/ethnicity, education, annual household income, number of previous live births, marital status, insurance coverage during pregnancy, and state of residence (obtained from the linked birth certificate). We also examined data on year of delivery and infant age.

PRAMS asks about the average number of cigarettes smoked per day during the 3 months before pregnancy, during the last 3 months of pregnancy, and after delivery. Postpartum smoking status was categorized into 4 mutually exclusive categories: 1) nonsmoker before and during pregnancy and postpartum, 2) quit during pregnancy and remained quit postpartum, 3) quit during pregnancy and relapsed postpartum, and 4) smoked during pregnancy and postpartum. We also examined PRAMS data on the mother’s self-reported attendance at the infant’s first-week health care visit, any well-baby visit at 2, 4, or 6 months of age, the mother’s postpartum check-up (available for Arkansas and New Jersey only), and participation in WIC.

Data on 42,208 women were available for analysis. We excluded women who lacked data on smoke-free–home rules (n = 673 [1.6%]) or smoking status (n = 1,121 [2.7%]); the percentage of respondents who lacked data did not vary by state. The final sample for bivariate analyses included 41,535 women (98.4%). For all other variables, the percentage of respondents who lacked data ranged from 0.02% (age) to 7.4% (income). The final multivariable model included 36,804 (87.2%) women.

### Statistical analysis

We calculated the overall prevalence of women reporting complete, partial, and no rules. For each state, we calculated the prevalence of complete rules by year, tested for linear trends by using the Cochran–Mantel–Haenszel test for trend at *P* < .05, and calculated the percentage change in prevalence of complete rules for each state from 2004 to 2008. We calculated the prevalence and 95% confidence intervals (CIs) of women reporting complete rules by potential correlates (postpartum smoking status, demographic characteristics, year of infant delivery, and state of residence) and examined differences by using χ^2^ tests at *P* < .05. We modeled the associations between having a complete rule and potential correlates while controlling for all other variables. For the model, we combined the categories of partial and no rules because of the small percentage of women who reported no rules, the demographic similarities of the 2 groups, and evidence that partial rules do not offer protection from secondhand smoke exposure (8). We calculated adjusted prevalence ratios (APRs) and 95% CIs by using multivariable logistic regression as described by Bieler et al (10). Because we had an adequate sample size, we examined all potential correlates of smoke-free–home rules in the final multivariable model. Selection of potential correlates was based on associations with smoking and secondhand smoke exposure (2). Information on other smokers in the household was unavailable. We calculated the percentage of women who had partial and no rules by postpartum smoking status. We calculated the prevalence and 95% CIs of attendance at the infant’s first-week health care visit, any well-baby visit, maternal postpartum check-up, and WIC participation among women who reported partial or no rules. Analyses were conducted from October 2010 through November 2011 by using SAS software version 9.2 (SAS Institute, Cary, North Carolina) and SUDAAN version 10.0.1 (Research Triangle Institute, Research Triangle Park, North Carolina) to account for the complex survey design of PRAMS.

## Results

During 2004–2008, among the 5 study states, 94.6% of women with infants had complete smoke-free–home rules, 4.6% had partial rules, and 0.8% had no rules (Table 1). Prevalence of complete rules varied by state and ranged from 85.4% (Arkansas) to 98.1% (Oregon). Prevalence increased significantly in 3 states (Arkansas, Maine, New Jersey) from 2004 to 2008 (Figure); also during that time, the proportion increase in prevalence ranged from 0.7% in Washington to 11.9% in Arkansas.

**Table 1:** Prevalence of Smoke-Free–Home Rules Among Women With Infants (n = 41,535), Overall and by Characteristic, PRAMS 2004–2008^a^

Characteristic	Complete Rule, % (95% CI) (n = 38,084	Partial Rule, % (95% CI) (n = 2,883)	No Rule, % (95% CI) (n = 568)	*P* Value^b^
**Total**	94.6 (94.4–94.9)	4.6 (4.3–4.8)	0.8 (0.7–0.9)	
**Postpartum smoking status** (n = 40,586)				
Nonsmoker during pregnancy and postpartum	97.0 (96.8–97.3)	2.5 (2.3–2.7)	0.5 (0.4–0.6)	<.001
Quit and remained quit postpartum	95.1 (93.6–96.3)	4.5 (3.4–6.1)	0.3 (0.2–0.5)	
Quit and relapsed postpartum	89.2 (87.3–90.9)	9.1 (7.6–10.9)	1.6 (1.0–2.7)	
Smoker during pregnancy and postpartum	78.6 (77.1–80.1)	18.4 (17.0–19.8)	3.0 (2.5–3.6)	
**Age, y** (n = 41,528)				
<20	87.6 (86.1–88.9)	10.9 (9.6–12.3)	1.6 (1.1–2.1)	<.001
20–24	91.3 (90.6–92.0)	7.4 (6.7–8.1)	1.3 (1.1–1.6)	
25–34	96.4 (96.0–96.7)	3.0 (2.8–3.3)	0.6 (0.5–0.7)	
≥35	97.0 (96.4–97.5)	2.7 (2.2–3.3)	0.3 (0.2–0.5)	
**Race/ethnicity** (n = 41,507)				
Non-Hispanic white	94.6 (94.3–95.0)	4.5 (4.2–4.9)	0.9 (0.7–1.0)	<.001
Non-Hispanic black	86.5 (85.3–87.6)	12.0 (10.9–13.1)	1.5 (1.1–2.0)	
Hispanic	97.3 (96.8–97.7)	2.3 (1.9–2.8)	0.4 (0.2–0.6)	
Other	96.6 (95.9–97.1)	2.8 (2.3–3.5)	0.6 (0.4–0.9)	
**Education, y** (n = 41,206)				
<12	90.5 (89.5–91.3)	8.0 (7.3–8.9)	1.5 (1.2–1.9)	<.001
12	91.5 (90.8–92.1)	7.2 (6.6–7.8)	1.3 (1.1–1.6)	
>12	97.5 (97.2–97.7)	2.2 (2.0–2.5)	0.3 (0.2–0.4)	
**Annual income, $** (n = 38,759)				
<10,000	88.5 (87.6–89.4)	9.9 (9.0–10.7)	1.7 (1.3–2.0)	<.001
10,000–14,999	90.8 (89.6–91.9)	7.9 (6.9–9.1)	1.3 (0.9–1.8)	
15,000–19,999	92.3 (90.8–93.6)	6.7 (5.5–8.2)	1.0 (0.6–1.6)	
20,000–24,999	93.3 (92.0–94.4)	5.9 (4.9–7.1)	0.8 (0.5–1.4)	
25,000–34,999	95.1 (94.2–95.9)	3.9 (3.2–4.8)	0.9 (0.6–1.5)	
35,000–49,999	97.2 (96.5–97.8)	2.2 (1.7–2.8)	0.6 (0.3–0.9)	
≥50,000	98.3 (98.0–98.6)	1.5 (1.2–1.8)	0.2 (0.1–0.3)	
**Previous live births, no.** (n = 41,287)				
0	94.5 (94.1–95.0)	4.7 (4.3–5.1)	0.7 (0.6–0.9)	.50
≥1	94.6 (94.2–94.9)	4.6 (4.3–4.9)	0.8 (0.7–1.0)	
**Marital status** (n = 41,512)				
Married	97.1 (96.8–97.3)	2.4 (2.2–2.7)	0.5 (0.4–0.6)	<.001
Unmarried	89.8 (89.1–90.4)	8.8 (8.3–9.4)	1.4 (1.2–1.7)	
**Insurance coverage during pregnancy** (n = 41,017)				
Medicaid	90.3 (89.7–90.9)	8.3 (7.7–8.9)	1.4 (1.2–1.7)	<.001
Other	97.3 (97.1–97.6)	2.3 (2.1–2.6)	0.4 (0.3–0.5)	
Uninsured	94.2 (93.3–95.1)	4.7 (3.9–5.6)	1.1 (0.7–1.5)	
**Infant delivery year** (n = 41,535)				
2004	92.6 (91.9–93.2)	6.3 (5.7–6.9)	1.2 (0.9–1.5)	<.001
2005	94.0 (93.3–94.6)	5.1 (4.6–5.8)	0.9 (0.7–1.2)	
2006	95.0 (94.4–95.5)	4.4 (3.9–5.0)	0.6 (0.4–0.8)	
2007	95.6 (95.1–96.1)	3.8 (3.3–4.3)	0.6 (0.4–0.9)	
2008	95.7 (95.0–96.2)	3.6 (3.1–4.2)	0.8 (0.5–1.1)	
**Infant age, mo** (n = 40,558)				
<3	95.9 (95.3–96.4)	3.6 (3.1–4.2)	0.5 (0.4–0.7)	<.001
3–5	94.4 (94.1–94.8)	4.7 (4.4–5.1)	0.8 (0.7–1.0)	
>5	93.2 (92.2–94.1)	5.6 (4.9–6.6)	1.1 (0.8–1.6)	
**State** (n = 41,535)				
Arkansas	85.4 (84.4–86.2)	12.3 (11.5–13.2)	2.3 (2.0–2.7)	<.001
Maine	92.4 (91.5–93.1)	6.6 (5.9–7.4)	1.0 (0.8–1.4)	
New Jersey	94.5 (94.0–95.0)	4.8 (4.3–5.2)	0.7 (0.6–0.9)	
Oregon	98.1 (97.6–98.5)	1.6 (1.2–2.0)	0.3 (0.2–0.6)	
Washington	97.2 (96.6–97.7)	2.4 (1.9–2.9)	0.4 (0.3–0.7)	

Abbreviations: PRAMS, Pregnancy Risk Assessment Monitoring System; CI, confidence interval.

^a^ PRAMS data from 5 states (Arkansas, Maine, New Jersey, Oregon, and Washington). All n’s are unweighted. Smoke-free–home rules were determined by asking the following question: “Which of the following statements best describes the rules about smoking inside your home now?” Respondents were categorized as having complete rules (“no one was allowed to smoke anywhere inside my home”); partial rules (“smoking was allowed in some rooms or at some times”); or no rules (“smoking was permitted anywhere inside my home”).

^b^ Determined by χ^2^ test.

**Figure:**
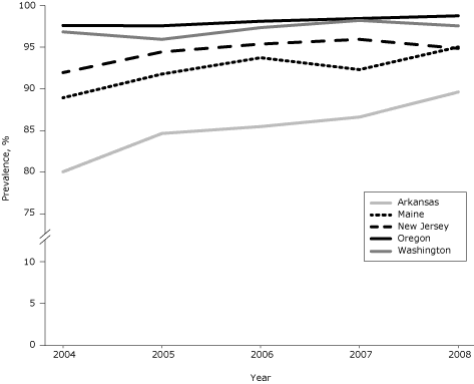
Prevalence of complete smoke-free–home rules among women with infants in 5 states, Pregnancy Risk Assessment Monitoring System, 2004–2008. Prevalence increased significantly in Arkansas, Maine, and New Jersey (Cochran–Mantel–Haenszel test for trend). **Table:** 

State	2004	2005	2006	2007	2008
**Arkansas**	**80.1**	**84.7**	**85.5**	**86.6**	**89.6**
**Maine**	**89.0**	**91.8**	**93.8**	**92.3**	**95.0**
**New Jersey**	**92.0**	**94.4**	**95.4**	**96.0**	**94.9**
**Oregon**	**97.6**	**97.6**	**98.1**	**98.4**	**98.8**
**Washington**	**96.8**	**96.0**	**97.3**	**98.2**	**97.6**

In bivariate analysis, we found significant differences in the prevalence of complete, partial, and no rules for all variables except number of previous live births (Table 1). Overall, although the prevalence of complete rules was generally high (>78%), we found disparities. The lowest prevalence of complete rules was among women who smoked during pregnancy and postpartum (78.6%). Prevalence was also low among these groups: women younger than 20 years (87.6%), non-Hispanic blacks (86.5%), women who had fewer than 12 years of education (90.5%), women who had an annual household income of less than $10,000 (88.5%), unmarried women (89.8%), women enrolled in Medicaid during pregnancy (90.3%), and women who had infants aged 5 months or older (93.2%).

In the adjusted model, all variables except maternal age, previous live birth, and infant age were independently associated with having complete smoke-free–home rules; however, most APRs were close to 1.0 (range, 0.90–1.03) (Table 2). We found the strongest association with smoking status: women who smoked during and after pregnancy were less likely than nonsmokers to have complete rules (APR, 0.90; 95% CI, 0.89–0.92).

**Table 2:** Complete Smoke-Free–Home Rules Among Women With Infants (n = 36,084), by Characteristic, PRAMS 2004–2008^a^

Characteristic	Adjusted Prevalence Ratio^b^ (95% CI)
**Postpartum smoking status**	
Nonsmoker during pregnancy and postpartum	1 [Reference]
Quit and remained quit postpartum	0.99 (0.97–1.00)
Quit and relapsed postpartum	0.96 (0.95–0.97)
Smoker during pregnancy and postpartum	0.90 (0.89–0.92)
**Age, y**	
<20	0.98 (0.97–1.00)
20–24	0.99 (0.98–1.01)
25–34	1.00 (0.99–1.01)
≥35	1 [Reference]
**Race/ethnicity**	
Non-Hispanic white	1 [Reference]
Non-Hispanic black	0.98 (0.97–0.99)
Hispanic	1.03 (1.02–1.04)
Other	0.99 (0.98–1.01)
**Education, y**	
<12	0.98 (0.97–0.99)
12	0.99 (0.98–0.99)
>12	1 [Reference]
**Annual income, $**	
<10,000	0.96 (0.95–0.97)
10,000–14,999	0.97 (0.95–0.98)
15,000–19,999	0.97 (0.96–0.99)
20,000–24,999	0.97 (0.96–0.98)
25,000–34,999	0.98 (0.97–0.99)
35,000–49,999	1.00 (0.99–1.01)
≥50,000	1 [Reference]
**Previous live birth, no.**	
0	1 [Reference]
≥1	1.00 (0.99–1.00)
**Marital status**	
Married	1.01 (1.00–1.02)
Unmarried	1 [Reference]
**Insurance coverage during pregnancy**	
Medicaid	1 [Reference]
Other	1.01 (1.00–1.02)
Uninsured	1.01 (1.00–1.02)
**Infant delivery year**	
2004	0.97 (0.96–0.98)
2005	0.98 (0.98–0.99)
2006	0.99 (0.99–1.00)
2007	1.00 (0.99–1.01)
2008	1 [Reference]
**Infant age, mo**	
<3	0.99 (0.98–1.01)
3–5	1.00 (0.99–1.01)
>5	1 [Reference]
**State**	
Arkansas	0.93 (0.92–0.94)
Maine	0.96 (0.96–0.97)
New Jersey	0.95 (0.94–0.95)
Oregon	1 [Reference]
Washington	0.99 (0.98–1.00)

Abbreviations: PRAMS, Pregnancy Risk Assessment Monitoring System; CI, confidence interval.

^a^ PRAMS data from 5 states (Arkansas, Maine, New Jersey, Oregon, and Washington). Smoke-free–home rules were determined by asking the following question: “Which of the following statements best describes the rules about smoking inside your home now?” Respondents were categorized as having complete rules (“no one was allowed to smoke anywhere inside my home”); partial rules (“smoking was allowed in some rooms or at some times”); or no rules (“smoking was permitted anywhere inside my home”).

^b^ Logistic regression model adjusted for postpartum smoking status, age, race/ethnicity, education, income, previous live birth, marital status, insurance coverage during pregnancy, infant delivery year, infant age, and state.

Among women who had partial or no rules, 44% were nonsmokers during pregnancy and postpartum, and 42% were smokers during pregnancy and postpartum. Among women who had partial or no rules, 80.2% attended the infant’s first-week health care visit, 96.7% attended at least 1 well-baby visit, and 78.4% attended the postpartum check-up (Table 3). Two-thirds (66.6%) reported WIC participation.

**Table 3:** Attendance at Health Care Visits and Participation in WIC Among Women With Infants Who Reported Partial or No Smoke-Free–Home Rules (n = 3,451), PRAMS, 2004–2008^a^

Characteristic	No. of Respondents (Unweighted)	% (95% CI)
First-week infant health care visit	3,181	80.2 (78.0–82.2)
Any well-baby visit (at 2, 4, or 6 months)	3,186	96.7 (95.6–97.5)
Maternal postpartum visit^b^	2,538	78.4 (76.1–80.5)
WIC participation	3,432	66.6 (64.0–69.1)

Abbreviations: WIC, Special Supplemental Nutrition Program for Women, Infants, and Children; PRAMS, Pregnancy Risk Assessment Monitoring System; CI, confidence interval.

^a^ PRAMS data from 5 states (Arkansas, Maine, New Jersey, Oregon, and Washington). Smoke-free–home rules were determined by asking the following question: “Which of the following statements best describes the rules about smoking inside your home now?” Respondents were categorized as having complete rules (“no one was allowed to smoke anywhere inside my home”); partial rules (“smoking was allowed in some rooms or at some times”); or no rules (“smoking was permitted anywhere inside my home”).

^b ^Question on maternal postpartum visit asked only in Arkansas and New Jersey.

## Discussion

To our knowledge, this is the first study to examine smoke-free–home rules in a population-based sample of women with infants in the United States. A high percentage of women reported having complete smoke-free–home rules. This estimate is higher than the estimate found in the 2006–2007 Tobacco Use Supplement to the Current Population Survey; in this survey of US households with children aged 17 years or younger, 83.9% of households had complete smoke-free–home rules (11). We observed a significant increase in the prevalence of smoke-free–home rules in 3 of the 5 states in our analysis, although the prevalence was high for all study states. This increase may reflect differences in the presence of state or local laws banning smoking in public spaces. Several studies have found increases in the adoption of smoke-free–home rules after comprehensive smoke-free laws were implemented (12-14). During the study period, New Jersey in 2006 and Washington in 2005 implemented comprehensive state laws eliminating smoking in public places and workplaces, including restaurants and bars; Maine had a law in place making restaurants and bars smoke-free at the start of the study period (2004) but did not eliminate smoking in private workplaces until 2009; Oregon did not completely eliminate smoking in private workplaces, restaurants, and bars until 2009; and Arkansas implemented partial smoking restrictions in 2006, which made private workplaces smoke-free but exempted restaurants and bars (6,15). Our findings show that efforts by public health practitioners and clinicians to increase awareness of the health effects of secondhand smoke on infants might have been successful. Because public smoking restrictions have been associated with increases in smoke-free–home rules and decreases in smoking prevalence (12-14,16), we suspect that the prevalence of complete smoke-free–home rules remains high or may have increased in these states since 2008; however, follow-up is needed to assess these trends.

Although the prevalence of partial and no rules was low, an estimated 75,000 women with infants in these study states are living in homes that do not have a complete smoke-free–home rule, and of these women, half are current smokers, likely exposing their infant to second- and thirdhand smoke (ie, tobacco smoke that remains in clothes, hair, and surroundings after a cigarette is extinguished) (17). The characteristics of women with infants who had a partial or no rule (current smokers, non-Hispanic black women, and women who have <12 years of education) in our study were consistent with characteristics of women in other studies that have examined smoke-free–home rules among households with children of any age (18-20). Additionally, 44% of women who had partial or no rules were nonsmokers in our study. These women may share their households with partners, other relatives, or guests who smoke in the home (21). In our study, almost all women who had partial or no rules attended infant well-baby visits, and more than two-thirds of these women attended a postpartum checkup or participated in WIC. A woman’s visit to her prenatal care provider may end within 6 weeks after delivery, so well-baby visits or participation in other programs, such as WIC, offer repeated opportunities for providers to ask women and their partners about tobacco use and exposure to second- and thirdhand smoke; providers should advise all families to make their homes, cars, and other environments completely smoke-free (22). Furthermore, systems-based changes — entering data on secondhand smoke exposure into electronic medical records or reimbursing for counseling on tobacco exposure, for example — could help reduce barriers to intervention among providers (23).

This study has several limitations. First, the presence of a smoke-free–home rule was self-reported by the mother, so prevalence could have been overestimated. Also, we have no information on whether these rules are enforced. However, other studies have found that parental report of home rules correlates with biomarkers for exposure to secondhand smoke among children (24,25). Second, we were not able to assess the presence of other smokers living in the household, which is an important predictor of secondhand smoke exposure in the home. Third, our findings are generalizable only to women with infants in the study states, and therefore may not be generalizable to the entire United States. Although the response rates ranged from 71% to 82%, the PRAMS methodology weights data to account for nonresponse. A potential selection bias may result if the weighting procedure does not fully address nonresponse. Fourth, the regression analysis combined data sets across multiple years to increase sample size. There exists the small possibility that mothers who had multiple pregnancies in these states from 2004 through 2008 were represented more than once. Because data from different years are not linked by mother, we were unable to account for correlation by mother in our analysis, but the likelihood of the women being sampled twice is low. We also included state and year in the model to reduce potential confounders of using a combined data set. Finally, PRAMS does not ask about smoke-free rules in other settings where infants may be exposed to secondhand smoke, such as vehicles, day care, or school. However, infants spend most of their time at home, and home is the primary source of secondhand exposure for this age group (2).

A high percentage of women with infants in this study reported a complete smoke-free–home rule, suggesting successful public health and clinical efforts to educate parents about the risk of secondhand smoke exposure to infants. Efforts are still needed to reach small groups of women, including current smokers, who are least likely to have complete smoke-free–home rules. Additionally, counseling women about the health risks of secondhand smoke among children and continuing to promote the adoption of smoke-free–home rules at well-baby visits and obstetric/gynecologic visits and in other programs, such as WIC, may help to further reduce secondhand smoke exposure among infants and to improve short- and long-term infant health outcomes.
